# Hypnotic susceptibility and affective states in bipolar I and II disorders

**DOI:** 10.1186/s12888-017-1529-2

**Published:** 2017-11-09

**Authors:** Bingren Zhang, Jiawei Wang, Qisha Zhu, Guorong Ma, Chanchan Shen, Hongying Fan, Wei Wang

**Affiliations:** Department of Clinical Psychology and Psychiatry/ School of Public Health, Zhejiang University College of Medicine, Hangzhou, Zhejiang 310058 China

**Keywords:** Bipolar I and II disorders, Depression, Hypomania, Hypnotic susceptibility, Mania

## Abstract

**Background:**

Highly hypnotizable individuals have impaired executive function, elevated motor impulsivity and increased emotional sensitivity, which are sometimes found in bipolar disorder patients. It is then reasonable to assume that certain aspects of hypnotic susceptibility differ with the types of bipolar disorder.

**Methods:**

The Stanford Hypnotic Susceptibility Scale: Form C (SHSS:C) test, the Mood Disorder Questionnaire (MDQ), the Hypomanic Checklist-32 (HCL-32) and the Plutchick-van Praag Depression Inventory (PVP) were applied to 62 patients with bipolar I disorder, 33 bipolar II disorder, and 120 healthy volunteers.

**Results:**

The passing rate of the SHSS:C ‘Moving hands apart’ item was higher in bipolar I patients than in controls, whereas for ‘Mosquito hallucination’ the rate was lower. Bipolar I and II patients scored significantly higher on MDQ, HCL-32 and PVP scales than controls. The passing rates of ‘Mosquito hallucination’ in controls, ‘Arm rigidity’ in bipolar I, and ‘Age regression’ in bipolar II predicted the respective MDQ scores.

**Conclusion:**

In contrast to cognitive suggestions, bipolar I patients followed motor suggestions more often under hypnosis. Furthermore, both bipolar disorder patients and healthy volunteers demonstrated associations between mania levels and certain hypnotic susceptibility features. Our study aids in better understanding the altered conscious states in bipolar disorders, and encourages the use of related psychotherapy for these patients.

## Background

Hypnotic susceptibility (or hypnotizability) is the ability of an individual to experience suggested alterations in physiology, sensations, emotions, thoughts, or behaviors during hypnosis [1], which is relatively stable throughout life [2]. Literature shows that hypnotic susceptibility is an important factor in cognitive hypnotherapy, though it cannot accurately predict treatment outcome [3]. However, hypno-psychotherapy is recommended in the treatment of emotional disorders, as it can reduce patients’ reactivity to internal or external triggers [4]. Studies have shown that people with personality traits such as extraversion [5], absorption [6], fantasy proneness [7], and openness to experience [8], are more easily hypnotized. Interestingly, high hypnotizables have been reported to be more impulsive than the lows, and often display non-planning and motor impulsivity [9]. Additionally, they have been reported to experience more emotional feelings [10], empathy [11], and emotional contagion, as well as poses a heightened ability to access affective events in a nonhypnotic context [12].

Some studies have shown that high hypnotizables experience more vivid mental-imagery [13, 14], although these results are not always reproducible [15]. Other studies have indicated that high hypnotizables have more intrusive thoughts [16], which require a stronger intervention on the individual’s psyche to produce relevant imagery [17, 18]. Interestingly, clinical disorders such as posttraumatic stress disorder [19] and dissociative disorder [20], which have emotional and intrusive symptoms, often display high hypnotizability. Mechanisms behind these phenomena are believed to fit into the dissociated-control theory, which explains that hypnosis causes a release of lower-level cognitive systems from the organization and control of higher-level integrating processes [21]. In this case, the release of lower-level activities might be linked with the impaired executive function of the frontal lobe in these pathologies [22].

Bipolar disorders often present with succeeding episodes of mania, depression, euthymia [23], and severe impairments in executive functions [24–27], including inhibitory control [28–30]. Bipolar I (BD I) and II (BD II) are the main types of bipolar disorders, the former having a higher prevalence of reckless activity, distractibility, psychomotor agitation, irritable mood and increased self-esteem [31], whereas the latter is characterized by a more chronic depressive course [32], often over-activated in goal-directed tasks [33]. Both BD I and BD II patients are impulsive [34], however, BD I is also characterized by higher levels of impulsive sensation seeking and borderline traits [35, 36], whereas BD II by higher neuroticism [37]. As these different affective or personality traits are associated with hypnotizability (for instance, the proven link between neuroticism and hypnotizability [38]), different types of bipolar disorder may have different hypnotic characteristics.

Both BD I and BD II patients have impaired executive functions [24] and such dysfunctions are associated with hypnotizability [15], thus, these patients might seldom experience vivid imagery or other forms of cognitive dissociation during hypnosis [13, 14]. Instead, both types of patients, particularly those with BD I, have insufficient metacognition [39] and high motor impulsivity [31, 34]. Studies have shown that these characteristics cause passive movements in individuals during hypnosis [9, 15, 40], manifested by increased physical dissociation and responsiveness to motor suggestions [41].

In the present study, hypnotic susceptibility of both BD I and BD II patients was tested. We used a widely-accepted tool, namely, the Stanford Hypnotic Susceptibility Scale: Form C (SHSS:C) [41], to measure responsiveness to hypnotic suggestions, as well as the Mood Disorder Questionnaire (MDQ) [42], the Hypomania Checklist-32 (HCL-32) [43], and the Plutchik-van Praag Depression Inventory (PVP) [44], to measure mania, hypomania and depression levels in our participants respectively. Based on previous findings, we hypothesized that (1) patients with bipolar disorder, especially BD I, are more easily motor- instead of cognitive-hypnotized and that (2) the ongoing affective states, measured by MDQ, HCL-32 and PVP, in patients are associated with their hypnotic susceptibility.

## Methods

### Participants

Overall, 62 patients with BD I (32 men and 30 women; aged 19.95 years ±1.80 S.D., range 18**–**27 years), 33 BD II (10 men and 23 women; aged 19.67 ± 1.76, range 18–26), and 120 university students as healthy volunteers (controls; 45 men and 75 women; aged 20.53 ± 2.44, range 18–32) were enrolled in the study. All participants were right-handed, without previous hypnosis experience. A semi-structured interview was performed with each healthy participant to ensure that they were not suffering from any psychiatric or neurological problem. All patients were diagnosed by an experienced psychiatrist (WW), according to the Diagnostic and Statistical Manual of Mental Disorders (DSM-5) criteria [23]. Recent computer tomography or magnetic resonance imaging scans conducted on patients displayed normal skulls, midlines, and parenchyma, including cerebella and brain stems. The patients were not currently requiring in-patient care, and were not suffering from dissociative identity disorder, personality disorders, posttraumatic stress disorder, drug/ alcohol abuse or schizophrenia. Moreover, participants declared to be free from any drug or alcohol for at least 72 h prior to the test.

The participants were submitted to the SHSS:C test, and were then asked to complete the MDQ, HCL-32, and PVP scales in a quiet room.

### Hypnotic susceptibility test

The SHSS:C [41] is a standardized 12-item measurement of an individual’s response to suggestions following a hypnotic induction, in which items are offered in order of increasing difficulty. As a measure of heterogeneous characteristics, it consists of motor suggestions, which are direct calls for performance, such as ‘Hand-lowering’ , involving the lowering of an outstretched hand while imagining holding a heavy weight; challenge suggestions, during which participants experience loss of arbitrary motor control, such as ‘Arm rigidity’ and ‘Arm immobilization’; and cognitive suggestions, leading to changes in perception, memory, and cognition, such as ‘Mosquito hallucination’ , ‘Posthypnotic amnesia’ , ‘Age regression’ , etc. This method was demonstrated to be valid [45, 46] and reliable [46] in several cultures, including Chinese.

Individual SHSS:C tests were performed under the guidance of one of the authors, who was blind to the grouping information of the participants. The procedure lasted approximately 45 min, starting with a unified interpretational set and eye-closure induction. Each suggestion carried out successfully, as judged by the hypnotist according to objective criteria, was noted as passed and counted as one point. Considering the heterogeneity of the measure, both the passing rate of all 12 items and the rate of each item were calculated, according to the percentage of participants who had passed the item(s).

### Questionnaire measures

A. The Mood Disorder Questionnaire (MDQ).

The MDQ consists of three parts [42], including 13 forced-choice (yes or no) questions to assess the presence of symptoms and behaviors related to mania or hypomania, one question to determine whether two or more symptoms have been experienced at the same time, and one question to determine the extent to which symptoms have caused functional impairment, based on a scale ranging from “no problems” to “serious problems”. The MDQ internal reliability was .79 and was demonstrated to be valid in a sample of Chinese individuals [47].

B. The Hypomania Checklist-32 (HCL-32).

The HCL-32 is a self-assessment instrument comprising 32 items for detecting hypomanic symptoms [44]. Individuals were instructed to answer forced-choice (yes or no) questions about emotions, thoughts, or behaviors, and to answer questions regarding their duration, impact on family, social and work life, or reactions elicited from peers. The HLC-32 internal reliability was .88 and was demonstrated to be valid in a sample of Chinese individuals [48].

C. The Plutchik-van Praag Depression Inventory (PVP).

The PVP consists of 34 items [44], each with three scale points (0, 1, 2) corresponding to increasing depressive tendencies. Subjects have “possible depression” if they score between 20 and 25, or “depression” if they score above 25. The PVP is a valid measure consistent with other similar tools [49], and its internal reliability was .94 in a sample of Chinese individuals [50].

### Statistical analysis

In the three groups, mean age and mean MDQ, HCL-32 and PVP scores were analyzed by one-way ANOVA. Once a group effect was detected, a post-hoc Bonferroni test was used. The gender distribution and the total scores of SHSS:C in the three groups were analyzed by Chi-Square test. Considering that each SHSS:C item measures a different aspect of hyponotizability [41], the passing rates of each item were also computed by Chi-Square test. Once a group effect was detected, a post-hoc Dunn’s test was used, and the Bonferroni correction was used to adjust the multiple analyses. Based on a previous study [51], we applied the multiple linear regression analysis (backward method) to explore the relationships between SHSS:C passing rates, MDQ, HCL-32 and PVP scales, considering the affective states as potential predictors for the passing rates. The alpha level of significance (*p*) was set at .05.

## Results

No significant difference was found among the three groups regarding either age (F [2, 212] = 2.65, mean square effect (MSE) = 12.62, *p* = .07) or gender (*χ* ^2^ = 5.03, *df* = 2, *p* = .08). The internal reliabilities of MDQ, HCL-32 and PVP in the current sample were .78, .87, .94 respectively. The mean MDQ scores were significantly different among the three groups (F [2, 212] = 134.22, MSE = 647.29, *p* < .01, η^2^ = .56), with BD I patients scoring significantly higher than BD II patients and controls (*p*s < .01). The mean HCL-32 scores were significantly different among the three groups (F [2, 212] = 110.44, MSE = 1263.65, *p* < .01, η^2^ = .51), with BD I and BD II patients scoring significantly higher than controls, and BD I higher than BD II (*p*s < .01). The mean PVP scores were also significantly different among the three groups (F [2, 212] = 99.86, *p* < .01, MSE = 3149.69, η^2^ = .49), with BD II patients scoring significantly higher than both BD I and controls, and BD I higher than controls (*p*s < .01) (Table 1).

**Table 1:** Scale scores (mean ± S.D.) of the Mood Disorder Questionnaire, the Hypomania Checklist-32, and the Plutchik-van Praag Depression Inventory in healthy volunteers (controls, *n* = 120), and patients with bipolar I (BD I, *n* = 62) and II (BD II, *n* = 33) disorders

	Controls	BD I	BD II
Mood Disorder Questionnaire	4.47 ± 2.66	9.84 ± 1.27*	4.24 ± 1.62^#^
Hypomania Checklist-32	15.09 ± 3.77	22.94 ± 1.77*	18.42 ± 4.13*,^#^
Plutchik-van Praag Depression Inventory	8.48 ± 4.89	12.74 ± 7.14*	24.03 ± 4.79*,^#^

*, *p* < .05 vs. controls; ^#^, *p* < .05 vs. BD I

The internal reliability of SHSS:C in the current sample was .63 (*N* = 215). There were no significant differences in the total score of SHSS:C between the three groups (*χ* ^2^ = 0.60, *df* = 2, *p* = .74). By contrast, when comparing the passing rates of individual SHSS:C items, there were significant group effects on ‘Moving hands apart’ (*χ* ^2^ = 6.68, *df* = 2, *p* = .04, η^2^ = .19) and on ‘Mosquito hallucination’ (*χ* ^2^ = 7.56, *df* = 2, *p* = .02, η^2^ = .22). Post-hoc test detected that BD I patients passed the item ‘Moving hands apart’ significantly more often (adjusted *p* = .03, OR = 0.31), and passed the item ‘Mosquito hallucination’ less often (adjusted *p* = .03, OR = 2.28) than controls (see Table 2). No other significant differences among items were found between groups. Regarding the associations between hypnotic susceptibility and affective states, the passing number of SHSS:C ‘Mosquito hallucination’ (beta, .24; B, 1.39; standardized error, .52) in controls (adjusted R^2^, .05), ‘Arm rigidity’ (beta, .35; B, 1.02; standardized error, .36) in BD I (adjusted R^2^, .11), and ‘Age regression’ (beta, .35, B, 1.70; standardized error, .82) in BD II (adjusted R^2^, .09) significantly predicted the MDQ scores.

**Table 2:** Numbers of participants passed (passing rate of) the items of the Stanford Hypnotic Susceptibility Scale: Form C in healthy volunteers (controls, *n* = 120), and patients with bipolar I (BD I, *n* = 62) and II (BD II, *n* = 33) disorders

	Controls	BD I	BD II	*χ* ^2^(*df*: 2)	*p*	η^2^
Hand lowering	97 (80.8%)	54 (87.1%)	28 (84.8%)	1.22	.55	.03
Moving hands apart	89 (74.2%)	56 (90.3%)a	25 (75.8%)	6.68	.04	.19
Mosquito hallucination	85 (70.8%)	32 (51.6%)a	18 (54.5%)	7.56	.02	.22
Taste hallucination	91 (75.8%)	49 (79.0%)	27 (81.8%)	0.62	.73	.02
Arm rigidity	83 (69.2%)	47 (75.8%)	26 (78.8%)	1.66	.44	.05
Dream	46 (38.3%)	24 (38.7%)	15 (45.5%)	0.55	.76	.02
Age regression	104 (86.7%)	52 (83.9%)	29 (87.9%)	0.11	.95	< .01
Arm immobilization	65 (54.2%)	37 (59.7%)	23 (67.7%)	2.64	.27	.08
Anosmia to ammonia	47 (39.2%)	22 (35.5%)	18 (54.5%)	3.05	.22	.10
Hallucinated voice	18 (15.0%)	5 (8.1%)	3 (9.1%)	2.17	.34	.06
Negative visual hallucination	48 (40.0%)	19 (30.6%)	10 (31.3%)	1.65	.44	.05
Posthypnotic amnesia	18 (15.0%)	5 (8.1%)	6 (18.2%)	2.41	.30	.07

## Discussion

To the best of our knowledge, this is the first study to test hypnotic susceptibility in patients with bipolar disorder. Both BD I and BD II patients displayed more pronounced emotional problems, as previously reported [35, 36, 48]. No differences were found between groups among SHSS:C total scores, however, when regarding the individual items, BD I patients possessed higher passing rates of SHSS:C ‘Moving hands apart’ and lower of ‘Mosquito hallucination’. Furthermore, certain SHSS:C items were associated with the mania levels of both patients and healthy volunteers.

The ‘Moving hands apart’ is a simple motor suggestion [41], and the higher passing rate recorded in BD I patients suggested that it was easier for these patients to engage in less cognitive-demanding tasks. With impaired executive function and metacognition [39, 52] and high levels of impulsivity [34], these patients moved impulsively when hypnotic instructions were given [9, 40]. Moreover, the impaired executive function and metacognition might underlie the lower passing rate of ‘Mosquito hallucination’ in BD I group. Indeed, this hypnotic item is a positive hallucination, and its induction requires greater utilization of fantasy and imagery [53], which strongly demands the involvement of executive functions [54, 55]. Therefore, their abnormal hypnotizability fits into the dissociated-control theory [21], and is explainable by the impaired frontal lobe executive function reported in BD I patients [22], partly accounting for their distractibility, psychomotor agitation [31] and problem-solving difficulty [56].

A common relationship between mania symptoms awake and altered states of consciousness induced by hypnosis [57] was replicated in both BD I and BD II groups, which were supported by the finding that patients with bipolar disorder display higher mood instability and higher levels of intrusive prospective imagery [18]. Specifically, ‘Arm rigidity’ , an item of physical dissociation, was associated with the MDQ score in BD I patients, indicating that the drive of the behavioral activation system was higher and was associated with mania in this disorder [58]. ‘Age regression’ , an item of consciousness dissociation, was associated with the MDQ score in BD II patients, consistent with the findings of pronounced childhood trauma and related dissociative symptoms, and the manic-related anxiety characteristic to this disorder [59, 60]. ‘Mosquito hallucination’ , often a manifestation of irritability, was associated with the MDQ score in healthy volunteers, which was partly in line with that the auditory hallucinations were regularly seen in bipolar disorder patients, especially during their manic episodes [61, 62].

However, certain limitations of the present study should be considered, for instance, the relatively small sample size, as well as the failure to record normal and disordered personality traits of the participants. The lower adjusted R^2^s in our study also imply that there were other factors contributing to the manic expressions in bipolar disorders. Nevertheless, we have found higher motor- but lower cognitive-suggestions under hypnosis in BD I patients, and mania levels were associated with different hypnotic susceptibility features in all three groups.

## Conclusion

BD I patients followed motor suggestions more often, unlike cognitive suggestions, under hypnosis, while both bipolar disorder patients and healthy volunteers demonstrated an association between mania levels and certain hypnotic susceptibility features. Our findings contribute to the understanding of emotional, cognitive and behavioral alterations in bipolar disorder patients, and encourage the incorporation of related psychotherapy in their treatment.

## Abbreviations

BD I: Bipolar I
BD II: Bipolar II
BD: Bipolar disorder
HCL-32: Hypomania Checklist-32
MDQ: Mood Disorder Questionnaire
PVP: Plutchik-van Praag Depression Inventory
SHSS:C: The Stanford Hypnotic Susceptibility Scale, Form C
